# Full-Field Thickness Measurement of Paint Sensors Using Pulsed Terahertz Waves

**DOI:** 10.3390/s25041213

**Published:** 2025-02-17

**Authors:** Dae-Hyun Han

**Affiliations:** Department of Carbon Convergence Engineering, Wonkwang University, 460 Iskander-ro, Iksan-si 54538, Jeonbuk-do, Republic of Korea; dhhan85@wku.ac.kr

**Keywords:** full field, imaging, terahertz waves, thickness, thin film

## Abstract

This study presents a method for measuring the thickness and adhesion status of paint sensors using pulsed terahertz (THz) waves. Traditional measurement techniques, such as optical, X-ray, ultrasonic (UT), eddy current, and mechanical methods, are prone to accuracy issues and potential sample damage, particularly when evaluating adhesion. The pulsed THz wave approach enables the high-resolution, nondestructive evaluation of both thickness and adhesion status. The analysis of pulsed THz wave reflections from the interfaces of the paint sensor enables accurate measurements of thickness and the detection of adhesion issues. Validation against traditional thickness gauges and UT devices demonstrates the superior performance of the THz-wave-based method, particularly for identifying significant changes in thickness and adhesion defects. Furthermore, a full-field visualization technique is developed to map thickness variations across the entire sensor surface, offering detailed insights into the sensor conditions. The THz-wave-based method represents a significant advancement in nondestructive testing, providing a precise and comprehensive analysis of paint sensors while overcoming the limitations of conventional techniques.

## 1. Introduction

The thicknesses of thin films significantly affect the performance and quality of semiconductors, displays, solar cells, and biosensors. Therefore, an accurate and efficient thin-film thickness measurement technique that does not damage the film is necessary [1,2]. However, existing thin-film thickness measurement methods possess the following limitations.

1. While optical methods [3,4,5] enable noncontact measurement, they are influenced by surface characteristics and may exhibit reduced accuracy with nonuniform thickness;
2. X-ray-based methods [6] pose radiation hazards, incur high costs, and have limited measurement ranges despite their effectiveness;
3. Ultrasonic (UT) methods [7,8,9] require impedance matching and may not accurately capture the adhesion status between the thin film and substrate;
4. Eddy current methods [10,11,12] are restricted by the type of material under inspection and may demonstrate decreased accuracy if the electrical properties fluctuate;
5. Mechanical methods can introduce measurement errors depending on the applied force and may cause damage based on the diameter of the probe.

These limitations indicate the need for a technique capable of simultaneously measuring both thickness and adhesion status without compromising accuracy or safety. To address this issue, this study proposes a method utilizing pulsed terahertz (THz) waves for thin-film thickness measurement [13] and adhesion status diagnosis. THz waves, which belong to the electromagnetic spectrum and have frequencies ranging from 0.1 to 10 THz, lie in the spectrum between light waves and radio waves, possessing both the penetration capabilities of radio waves and the directional properties of light waves. Given these properties, THz waves are particularly advantageous for non-destructive thin-film inspection, enabling both precise thickness measurement and adhesion analysis [14,15,16,17,18,19].

The advantages of using pulsed THz waves for measuring thin-film thickness are numerous:

1. Nondestructive method: THz waves penetrate various media, including air, without contact, preventing sample damage or contamination;
2. High-resolution method: THz waves precisely differentiate reflected signals from the thickness and interfaces of the thin film owing to a shorter wavelength and higher temporal resolution than ultrasound, eliminating the requirement for additional procedures such as impedance matching;
3. Multi-functional method: THz waves can simultaneously analyze adhesion status in addition to measuring thickness.

Smart paint sensors composed of ceramic and polymer composites [20,21,22] have attracted significant attention in various engineering applications. These thin-film sensors effectively detect impacts and vibrations through the piezoelectric effect while simultaneously utilizing the flexibility of polymer-based materials, facilitating their integration onto complex and curved surfaces. However, the thickness of these sensors plays a critical role in determining their sensitivity and frequency response, making precise thickness control essential [23,24,25,26,27]. Despite the importance of thickness in smart paint sensors, no prior studies have attempted to apply THz waves for this purpose. The proposed THz-based method introduces a non-contact approach for simultaneously measuring thickness and diagnosing interfacial adhesion, addressing the limitations of existing techniques.

This study aims to validate the feasibility of THz-based thin-film inspection by conducting comparative experiments with existing measurement methods, assessing both thickness and interfacial adhesion in smart paint sensors.

## 2. Test Setup for Measuring THz Waves and Thickness

### 2.1. Measurement of Pulsed THz Waves

**Figure 1:**
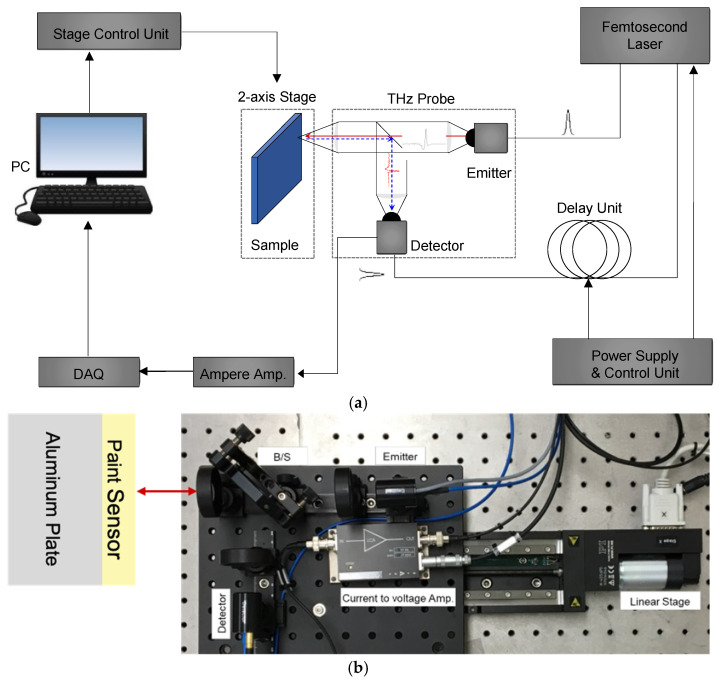
Test setup for the thickness measurement: (**a**) schematic and (**b**) actual.

A commercial fiber-coupled pulsed THz time-domain spectroscopy (THz-TDS) system (TERA K15, Menlo Systems Corp., Planegg, Germany) was used to acquire the THz waves reflected from the sample (Figure 1a). Although the THz-TDS system components were identical to those used in a previous study [28], the propagation paths of the THz waves were changed from the transmission to the reflection mode. The primary components of the TERA K15 included a femtosecond laser (T-Light, Menlo Systems Corp., Germany), optical delay unit (ODU, Menlo Systems Corp., Germany), photoconductive antenna for the emitter (TERA 15-TX-FC, Menlo Systems Corp., Germany), and detector (TERA 15-RX-FC, Menlo Systems Corp., Germany), as shown in Figure 1.

### 2.2. Thickness Measurement Methods

**Figure 2:**
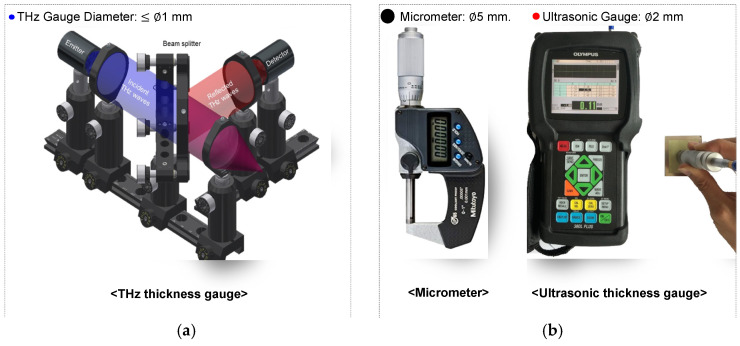
Thickness measurement methods: (**a**) proposed THz-based gauge and (**b**) commercial micrometer and ultrasonic gauge.

An experimental setup was designed to validate the proposed THz wave-based thickness measurement method against commercially used techniques (Figure 2). The THz-wave-based thickness measurement system (Figure 2a) employed a beam splitter to ensure that the THz waves emitted from the emitter were perpendicularly incident (θ = 0°) onto the sample. The detector then measured the THz waves reflected from the boundaries of the medium.

A thickness gauge and a UT thickness gauge were employed for comparison (Figure 2b). The contact-based thickness gauge requires considering the probe diameter according to the measurement target. This study used a thickness gauge (Digital Micrometer IP65, Mitutoyo, Tokyo, Japan), a 5 mm diameter probe, and a resolution of ±1 µm. The UT thickness gauge chosen was the Olympus 38DL Plus model, featuring a 2 mm diameter probe.

### 2.3. Fabrication of the Smart Paint Sensor

**Figure 3:**
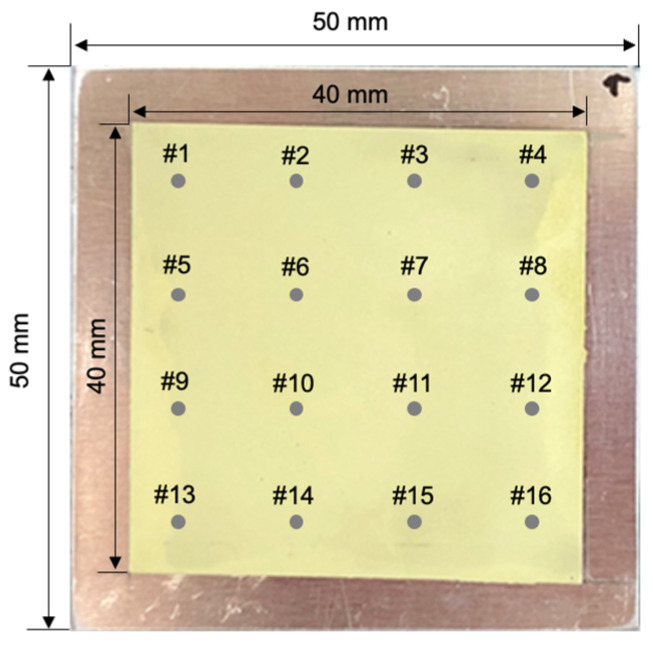
Smart paint sensor comprises PNN-PZT and epoxy resin.

The smart paint sensor comprised PNN-PZT ceramic powder and epoxy resin (Figure 3). PNN-PZT powder was synthesized using five raw materials: PbO (purity: 99.9%), Nb_2_O_5_ (purity: 99.9%), TiO_2_ (purity: 99.5%), NiO (purity: 99.99%), and ZrO_2_ (99.99%). Nb_2_O_5_ was added to produce a softer ceramic powder, while NiO reduced the sintering temperature to 1000 °C. All materials were procured from Sigma-Aldrich (Burlington, MA, USA). The PNN-PZT powder, epoxy resin, and hardener were mixed in a 1:1 weight ratio (12.2:87.8 volume fraction) to prepare the smart paint sensor. This mixture was stirred for 10 min in a vacuum desiccator to eliminate voids. Subsequently, the mixture was sprayed onto an aluminum plate with dimensions of 40 × 40 mm and cured in an oven at 80 °C. The paint sensor was subjected to a poling treatment after curing under optimal conditions, which included a poling electric field of 4 kV/mm and a poling time of 30 min at room temperature using a high-voltage amplifier (HCN-140, Fug, Germany) to activate the impact or vibration sensor [20]. Detailed information about the smart paint sensor is shown in Figure 3.

## 3. Experimental Principle

### 3.1. Principle of the Pulsed THz Wave Reflection and Thickness Calculation

The reflection of THz waves at interfaces where the medium changes, such as in smart paint sensors or delamination layers, can be explained by Snell’s law and the reflection coefficient.

(1) Snell’s law describes the refraction of light or electromagnetic waves as they pass through different media (Equation (1)).

(1) $$n_{1}\sin\left(\theta_{1}\right)=n_{2}\sin\left(\theta_{2}\right)$$

where $n_{1}$ and $n_{2}$ represent the refractive indices of the first and second media, respectively, and $\theta_{1}$ and $\theta_{2}$ denote the angles of incidence and refraction. In pulsed THz-TDS, the group refractive index characterizes the propagation speed of the pulse envelope, while the phase refractive index describes the phase velocity of individual frequency components. Due to the broadband nature of THz pulses, the dispersion effects are averaged, minimizing the difference between the group refractive index and the phase refractive index. Since the proposed method employs time-of-flight (TOF) analysis, the measured refractive index is inherently closer to the group refractive index. Therefore, in this study, all calculations were performed using the group refractive index.

(2) For the definition of the reflection coefficient, reflection signals are primarily generated at the boundary between different media. The reflection coefficient R represents the ratio of the intensity of the reflected wave to that of the incident wave and is defined as:

(2) $$R=({Z_{2}-Z_{1}/Z_{2}+Z_{1})}^{2}$$

where $Z_{1}$ and $Z_{2}$ correspond to the characteristic impedances of the first and second media, respectively. Light or electromagnetic waves reflect at the interfaces where the medium changes; these reflection signals are characterized by the reflection coefficient and are governed by Snell’s law. The reflection signals at the interface can be quantitatively described using these equations and principles.

**Figure 4:**
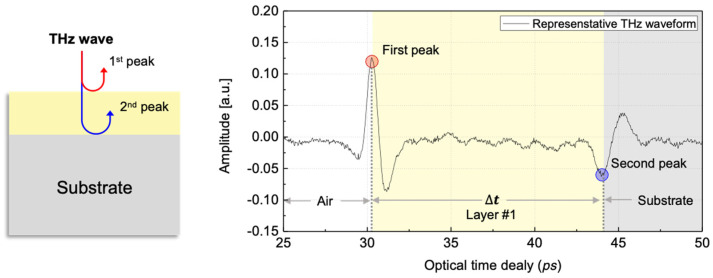
Representative THz wave at normal area.

The time-resolved detection scheme for pulsed THz-TDS is directly applicable to measuring the thickness of multi-layered samples. When pulsed THz waves are incident on an object, the reflected THz waveform consists of a series of pulses corresponding to reflections from the interfaces (Figure 4).

The thickness of a sample or defect can be calculated from the measured THz waves using the following equation:

(3) $$T_{sample}=\frac{∆t}{2}\times \frac{c}{n_{sample}}\times \cos\theta ,$$

where $T_{sample}$ represents the thickness of the sample, ∆t denotes the peak-to-peak time (Figure 4), *c* is the speed of light in air, $n_{sample}$ corresponds to the refractive index of the sample, and θ(=0°) represents the incident angle of the THz waves.

### 3.2. Thickness Calculation Using THz Waves

**Figure 5:**
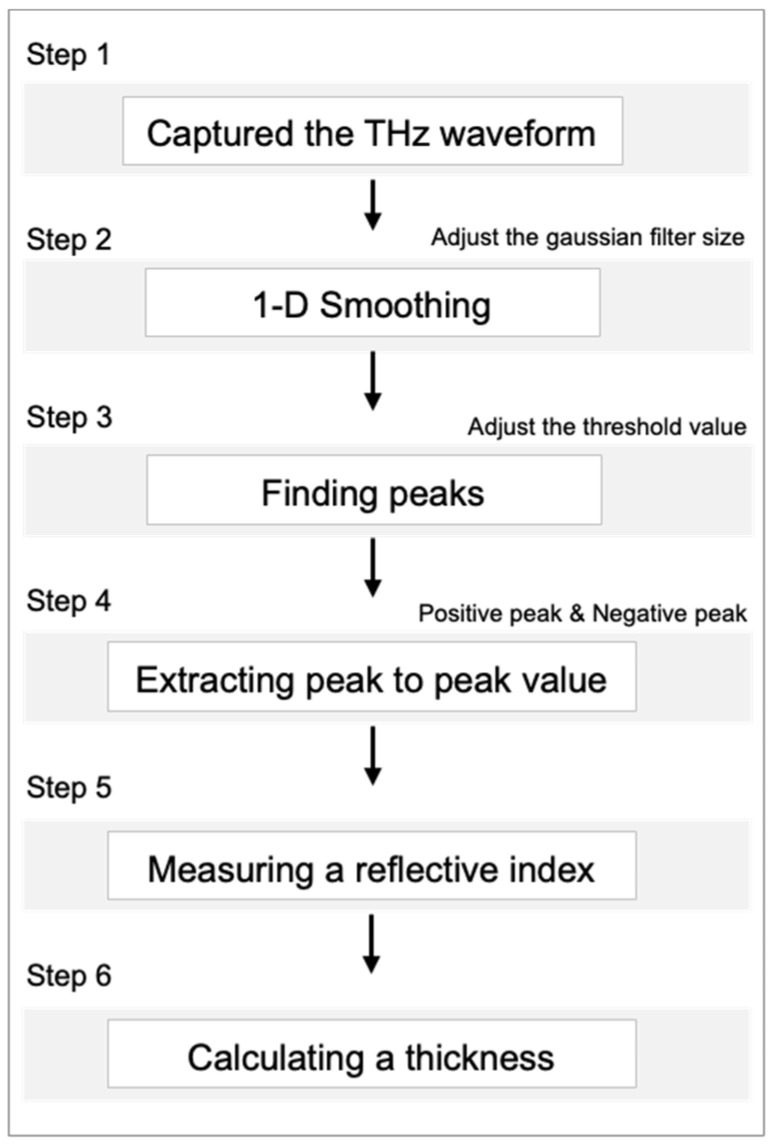
Overall process for calculating a thickness using THz wave.

**Figure 6:**
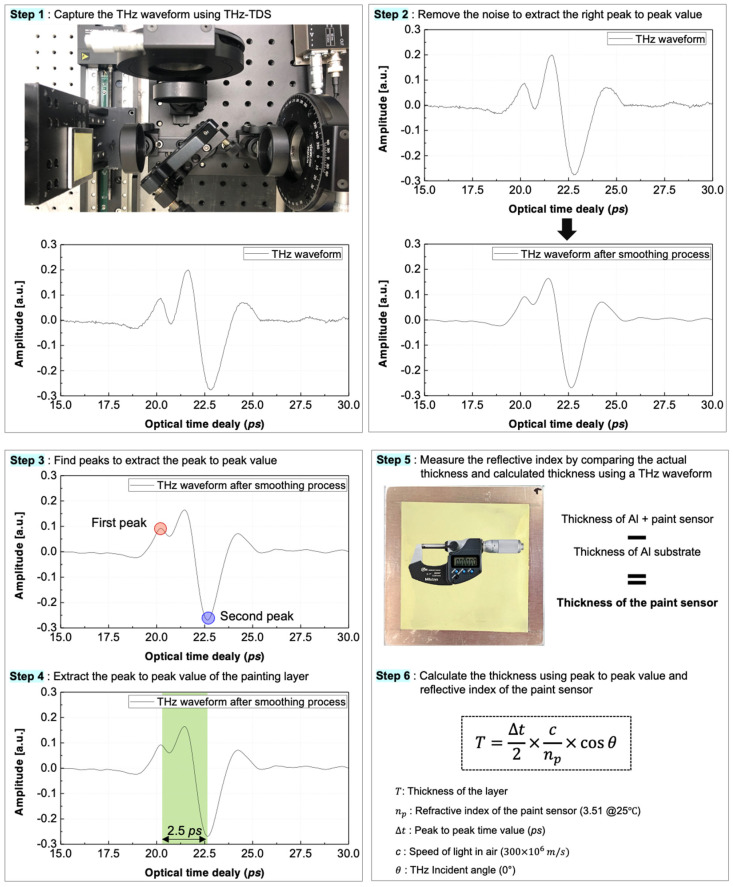
Detailed process for calculating a thickness using THz wave.

The THz waves measured in the reflection mode contained signals reflected at the interface of the medium. These reflected signals were measured from both the surface and backside of the paint sensor. The process involved identifying the peaks in the reflected signals at each position, which served as the basis for estimating the thickness (Figure 5 and Figure 6).

(1) For the THz wave measurement, reflected signals from the sample were measured using THz-TDS. Two and three peaks overlapped in normal regions and detachment areas, respectively;

(2) For noise filtering, noise in the measured signals was removed using a Gaussian filter to extract accurate reflection signals. To reduce the noise of the THz signal, we applied a Gaussian filter with a sigma value of three. Since the level of smoothing is determined by both the sigma value and the kernel size, we carefully selected these parameters to avoid significant distortion of the signal features. As shown in Figure 6, this smoothing process does not alter the position of the extrema. To further verify this, we compared the measured thickness values using a thickness gauge, confirming that the smoothing process did not introduce errors;

(3) For peak extraction, peaks were extracted from the THz waves reflected at the surface and backside of the paint sensor, facilitating the analysis of the intensity and temporal variations in the reflection signals;

(4) For the calculation of time delay values, time delay values between the extracted peaks were calculated to represent the time required for the reflection signals to pass through the paint sensor;

(5) For the measurement of paint sensor thickness for refractive index, the thickness of the paint sensor was measured using a thickness gauge, and its refractive index was calculated, enabling precise determination of the refractive index and thickness. In reflection mode, the reflected signals from both the surface and the backside of the layer were analyzed, allowing for the measurement of relative thickness variations without requiring prior knowledge of the exact group refractive index. By utilizing time-of-flight differences, the relative thickness was determined, ensuring the feasibility of the proposed method, even in the absence of direct calibration,

(6) For thickness estimation, the thickness of the paint sensor was estimated using the measured thickness and refractive index, facilitating the quantitative assessment of the thickness and adhesion status of the paint sensor.

### 3.3. Full-Field Thickness Image Method

**Figure 7:**
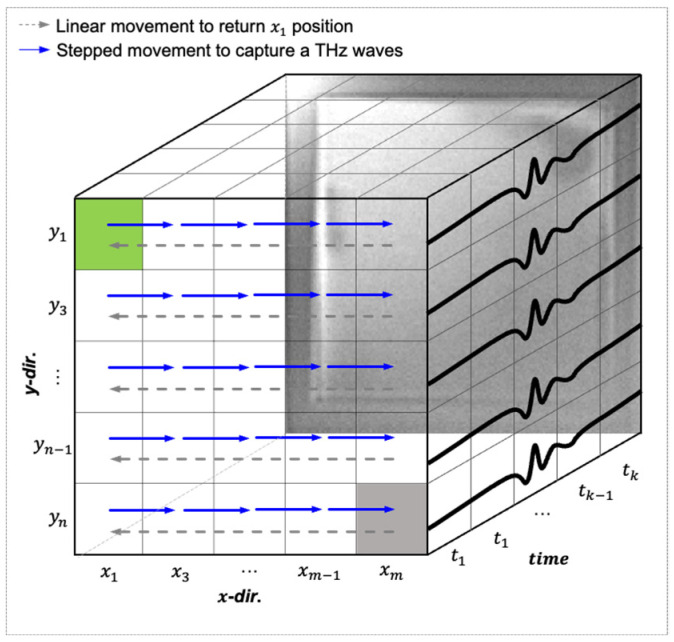
Reconstruction method from 1D to 3D THz wave for full-field image.

Figure 7 illustrates the reconstruction method from a 1D to a 3D THz wave structure for generating the full-field thickness image based on the raster-scanning method [29]. The THz wave corresponding to each raster pixel was acquired during the raster-scanning process, while the motorized *x-y* linear stage moved from the starting point ($x_{1},\;y_{1}$) to the endpoint ($x_{m},\;y_{1}$). Subsequently, the motorized *x-y* linear stage returned to the starting point ($x_{1},\;y_{1+i}$) for the next scan line without acquiring the THz wave. This process continued until the motorized *x-y* linear stage reached the termination point ($x_{m},\;y_{n}$). The thickness of the sample at each point was calculated following the method outlined in Section 3.2. The thickness map can be visualized by associating the thickness with the suitable x- and y-coordinates.

The full-field thickness visualization method proposed in a previous study [16] was employed in the time domain. Visualization in the time domain using a measured THz waveform is called THz pulse time-domain visualization (TTV). The visualization process (Figure 7) involved the following steps:

(1) THz waves were measured for each pixel in the inspection area;

(2) The motorized stage affixed to the sample moved in the x direction during THz wave measurement. It moved in the y direction upon reaching the endpoint before returning to the start point and continuing with the measurement;

(3) The scanned THz waves were reshaped into a three-dimensional (3D) matrix (*x*-axis, *y*-axis, time);

(4) Thickness calculations were processed as explained in Section 3.2;

## 4. Results

### 4.1. THz Waveform Results in Time Domains

**Figure 8:**
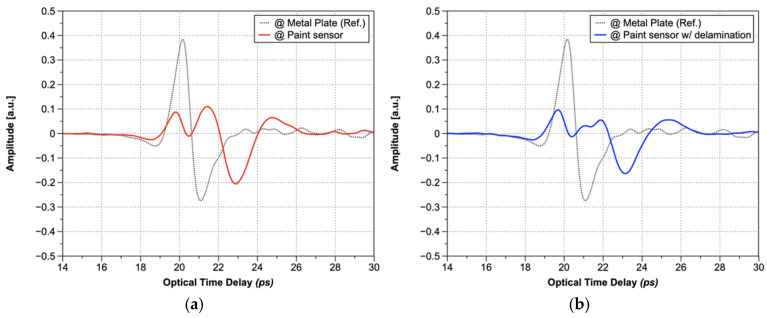
A-scan results: measured at (**a**) paint sensor and (**b**) paint sensor with delamination.

Figure 8a shows a representative THz wave in a normal area, whereas Figure 8b shows a representative THz wave in a delamination area. In well-adhered regions, reflection signals were observed only at the surface and the back surface (aluminum plate). Conversely, an additional reflection signal was detected at approximately 11 ps in the delaminated areas, corresponding to the air gap between the metal plate and the paint sensor (Figure 8b). This additional signal appeared alongside the reflections from the surface and back surface.

**Figure 9:**
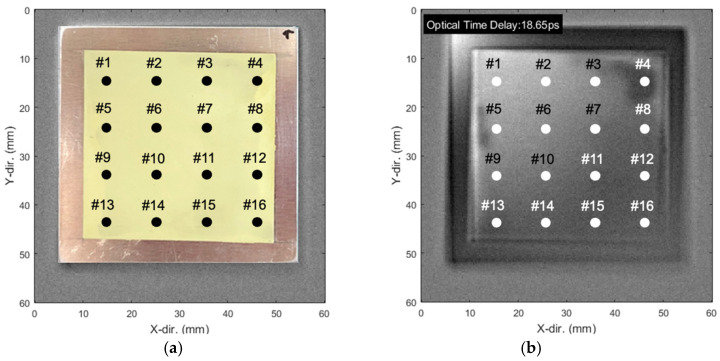
Smart paint sensor images: (**a**) optical image of the sensor with 16 sensing points and (**b**) full-field THz image showing the optical time delay distribution.

The THz waves measured at each pixel were analyzed to determine thickness and defect information, facilitating the construction of a full-field image. Figure 9 presents the visualization results of the full-field image of the sample. Figure 9a displays an optical image; Figure 9b depicts a phase-based image constructed from time-domain A-scan signals. In Figure 9b, the numbers are displayed in white to enhance readability against the grayscale background. Thicker and thinner regions are represented by bright and dark colors, respectively.

Locations from points #1 to #16 were selected for thickness measurement, considering the probe size of the thickness gauge with the largest measurement area. The progressive darkening observed from points #1 to #16 along the diagonal corresponds to the increasing distance between the THz detector and the specimen during the raster scan.

### 4.2. Thickness Measurement Results

Table 1 summarizes the measurement results obtained using the proposed THz method. For comparison, the results from ultrasonic and micrometer measurements are also included. The error bars were determined based on the range between the maximum and minimum values for each measurement point. The analysis of these error bars confirms the reproducibility of the proposed method, as the variations in the measured values remained within a small range (±0.001–0.002). Additionally, at certain measurement points (e.g., points 10, 11, and 14), no variation was observed, indicating stable and consistent measurements.

**Figure 10:**
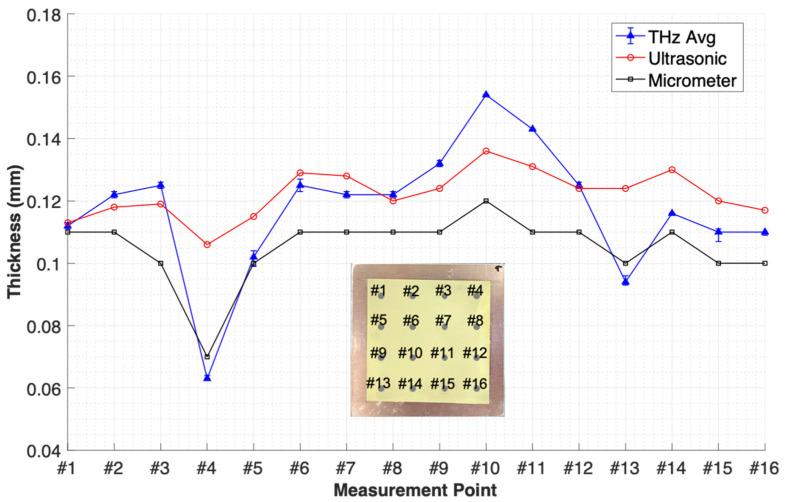
Thickness measurement results using various methods.

Figure 10 compares the thickness measurements of the smart paint sensor using the THz, micrometer, and UT methods at these points.

The trends observed with traditional methods and THz waves were consistent. However, significant discrepancies in accuracy were observed at point #4, where a rapid thickness change occurred due to differences in the probe diameters between each method. Both the UT method (2 mm) and the THz method (1 mm) accurately measured the rapid thickness change, whereas the thickness gauge (5 mm) reported a measurement 5 µm greater than the THz measurement. This discrepancy underscores the limitations of traditional methods because reducing the probe diameter of the thickness gauge to 1 mm, equivalent to the THz wave, could damage the polymer-based thin films.

The UT method, with the exception of measurement point #1, consistently measured the thickness to be approximately 10–20% thinner than the thickness gauge. This discrepancy arises from the lower spatial resolution of the UT method in the time domain compared to the THz method, as well as the challenges related to impedance matching when transmitting UT signals from the probe to the test specimen.

### 4.3. Full-Field THz Imaging Results

**Figure 11:**
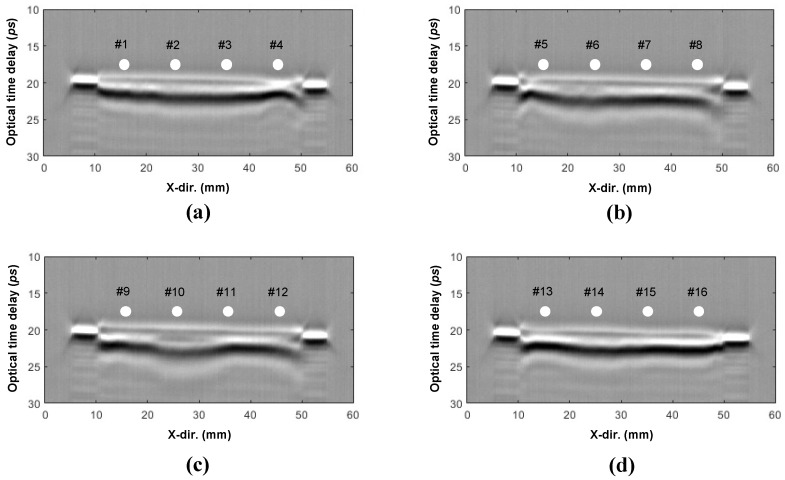
Cross-section images: (**a**) points 1–4, (**b**) points 5–8, (**c**) points 9–12, and (**d**) points 13–16.

**Figure 12:**
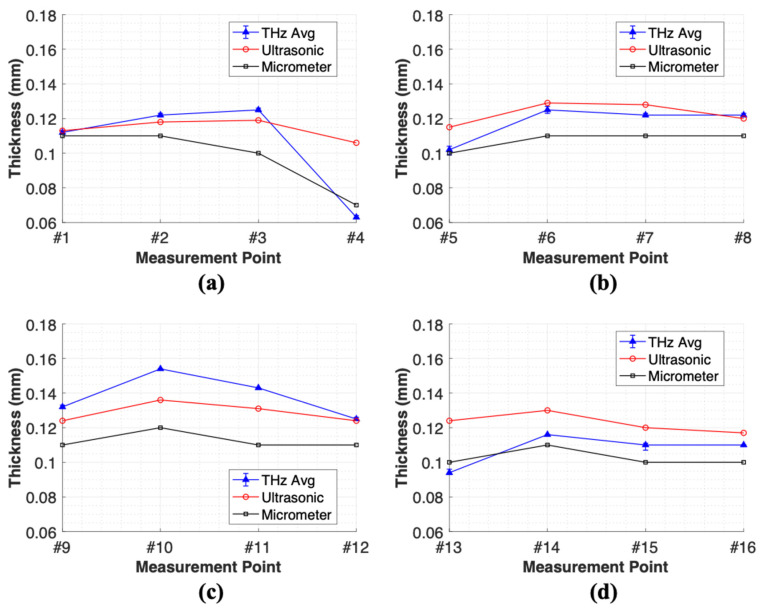
Thickness profile along measurement line: (**a**) points 1–4, (**b**) points 5–8, (**c**) points 9–12, and (**d**) points 13–16.

Figure 11 and Figure 12 present the results of the thickness measurements derived from the comparison and analysis of cross-sectional images of a smart paint sensor. Figure 11a–d display cross-sectional images, while Figure 12a–d show the thickness profiles calculated using peak-to-peak time delay values analyzed in the time domain. Figure 11a reveals an increase in film thickness from measurement points #1 to #3, followed by a sharp decrease at point #4. Both the THz and micrometer methods indicated an increase in thickness from points #1 to #3, which is consistent with the cross-sectional images (Figure 12a). However, the UT method showed a decreasing trend. The THz and UT methods accurately measured the drastically reduced thicknesses as 6.05 and 6.03 µm, respectively, at point #4, while the micrometer measured a thickness of 10.06 µm, 4.01 µm thicker than the THz method.

Figure 11b illustrates the cross-sectional images and thickness profiles corresponding to points #5 to #8. An increase in thickness was observed from points #5 to #6, a decrease at point #7, and a subsequent increase at point #8. The thickness profiles in Figure 12b show that the THz method and the micrometer provide similar results at points #6 to #8, where the thickness changes are minimal.

The micrometer demonstrated the lowest accuracy at point #5, where rapid thickness changes occurred. Delamination was observed at point #6, as indicated by the faint distinction between the bright and dark areas in the 21–22 ps range, reflecting signals from the air-layer boundary and signal attenuation. This phenomenon was more pronounced to the right of point #8.

Figure 11c shows the cross-sectional images for points #9 to #12, with the delamination most clearly observed at point #10 in the 21–22 ps range, indicating the presence of a delamination extending approximately 10 mm in the x direction. Figure 12c shows similar trends across all measurement methods, with the UT method consistently reporting the lowest thickness at each measurement position. Figure 11d displays the cross-sectional images for points #13 and #14; interlayer separation was observed at point #14. This outcome correlates with the interlayer separation at point #10, revealing an uneven thickness distribution.

Thickness gauges accurately measure gradual thickness changes. Conversely, the THz method provides accurate thickness measurements and can detect interlayer separation, irrespective of thickness variations and without requiring additional procedures, such as impedance matching or couplants. However, the UT method tends to measure thicknesses thinner than those measured by the thickness gauge, necessitating continuous impedance matching to improve accuracy. The smaller diameter of the measurement probe of the UT method resulted in a higher sensitivity to thickness variations than the thickness gauge.

## 5. Conclusions

This study proposed a novel approach for measuring the thickness and adhesion status of smart paint sensors using pulsed THz waves, addressing several limitations inherent in traditional measurement techniques and offering a nondestructive and highly accurate alternative. The key findings and implications are summarized as follows.

For nondestructive and high-resolution measurement, the application of pulsed THz waves enabled the nondestructive measurement of thickness and adhesion status, preserving sample integrity. The high temporal and spatial resolution of pulsed THz waves facilitated precise differentiation between reflection signals from various layers within the paint sensor.

For a comprehensive assessment of thickness and adhesion, unlike traditional methods that typically focus on thickness measurements, the THz-based approach provides simultaneous evaluation of both thickness and adhesion status, which is advantageous for applications requiring detailed assessment of thin films or multilayer paints.

For a comparative analysis with traditional techniques, the experimental data indicated that the accuracy of THz-based measurements was comparable to that of traditional thickness gauges and UT measuring instruments. The THz method demonstrated superior performance, especially in regions with abrupt thickness variations, where conventional methods often exhibit significant inaccuracies.

For full-field visualization capabilities, the full-field visualization of thickness variations and delamination across the entire sample area was achieved using raster scanning and 3D reconstruction techniques. This comparative mapping provided a clear and detailed visual representation of the sample conditions.

The advantage over traditional methods was that THz-based methods overcame several limitations associated with traditional techniques, such as the requirement for impedance matching in UT methods and the risk of sample damage in mechanical methods.

As to potential applications, this method holds significant promise for various industrial applications, including quality control of semiconductors, displays, solar cells, and biosensors. The ability to nondestructively assess thickness and adhesion status opens new avenues for advanced material characterization and monitoring.

The pulsed THz wave-based method for measuring thickness and adhesion status represents a major advancement in thin-film analysis. Its high accuracy, nondestructive nature, and comprehensive visualization capabilities make it an invaluable tool for a wide range of applications. Future research will focus on further refining this method and exploring its applicability to other types of thin films and coatings. Additionally, a more detailed analysis of the refractive index using effective medium theories, such as the Maxwell–Garnett model, will be conducted to further validate the experimental results.

## Figures and Tables

**Table 1:** Summary of the measurement results using the proposed THz method.

Method		Measurement Point (Unit: mm)															
		1	2	3	4	5	6	7	8	9	10	11	12	13	14	15	16
THz	Avg.	0.112	0.122	0.125	0.063	0.102	0.125	0.122	0.122	0.132	0.154	0.143	0.125	0.094	0.116	0.110	0.110
	Max	0.113	0.123	0.126	0.064	0.104	0.127	0.123	0.123	0.133	0.154	0.143	0.126	0.096	0.116	0.111	0.110
	Min	0.111	0.121	0.124	0.063	0.099	0.123	0.121	0.121	0.131	0.154	0.143	0.124	0.093	0.116	0.107	0.109
Ultrasonic		0.113	0.118	0.119	0.106	0.115	0.129	0.128	0.120	0.124	0.136	0.131	0.124	0.124	0.130	0.120	0.117
Micrometer		0.110	0.110	0.100	0.070	0.100	0.110	0.110	0.110	0.110	0.120	0.110	0.110	0.100	0.110	0.100	0.100

## Data Availability

Data sharing is not applicable to this article.
